# β1-Integrin Signaling is Essential for Lens Fiber Survival

**Published:** 2007-10-12

**Authors:** Andrew R. Samuelsson, Richard Belvindrah, Chuanyue Wu, Uli Müller, Willi Halfter

**Affiliations:** 1 Department of Neurobiology, University of Pittsburgh, 1402 E Biological Science Tower, Pittsburgh PA 15261; 2 Department of Cell Biology and Institute for Childhood and Neglected Disease, Scripps Research Institute, La Jolla, CA 92037; 3 Department of Pathology, 707 Scaife Hall, University of Pittsburgh, Pittsburgh PA 15261

**Keywords:** lens development, basement membrane, integrins, ILK, eye development

## Abstract

Integrins have been proposed to play a major role in lens morphogenesis. To determine the role of β1-integrin and its down-stream signaling partner, integrin linked kinase (ILK), in lens morphogenesis, eyes of WT mice and mice with a nestin-linked conditional knockout of β1-integrin or ILK were analyzed for defects in lens development. Mice, lacking the genes encoding the β1-integrin subunit (*Itgb1*) or ILK (*Ilk*), showed a perinatal degeneration of the lens. Early signs of lens degeneration included vacuolization, random distribution of lens cell nuclei, disrupted fiber morphology and attenuation and separation of the lens capsule. The phenotype became progressively more severe during the first postnatal week eventually leading to the complete loss of the lens. A more severe phenotype was observed in ILK mutants at similar stages. Eyes from embryonic day 13 β1-integrin-mutant embryos showed no obvious signs of lens degeneration, indicating that mutant lens develops normally until peri-recombination. Our findings suggest that β1-integrins and ILK cooperate to control lens cell survival and link lens fibers to the surrounding extracellular matrix. The assembly and integrity of the lens capsule also appears to be reliant on integrin signaling within lens fibers. Extrapolation of these results indicates a novel role of integrins in lens cell-cell adhesions as well as a potential role in the pathogenesis of congenital cataracts.

## Introduction

Lens formation begins with the induction and infolding of the epidermis by the eye vesicle to form a lens-shaped hollow ball of epithelial cells encased in an extra-cellular matrix (ECM) structure called the lens capsule. Whereas the anterior epithelial cells maintain a monolayer along the anterior surface of the lens, the posterior cells elongate and denucleate to become the primary lens fibers. A third population of epithelial cells located at the equatorial poles then elongates in the anterior and posterior directions along anterior epithelium and lens capsule substrates. These secondary fibers wrap around the primary lens fiber core forming onion-like growth shells and making connections with other fibers at the anterior and posterior seams. As secondary fibers mature they denucleate, disconnect from the lens capsule and are displaced to a more central location by younger fibers. Proliferation and differentiation of secondary lens fibers occur in a slow, but life-long manner with new generations of secondary fibers completing subsequent superficial growth shell layers (Rafferty and Rafferty, 1981; Zwaan and Kenyon, 1984; Bassnett and Winzenburger, 2003; Kuszak et al. 2004; Zelenka, 2004).

The molecular machinery that controls the development and maintenance of the lens is just beginning to be elucidated. The transcription factor Pax6 (Epstein et al. 1994; Azuma et al. 1999), and the growth factors FGF-2 (McAvoy et al. 1991; Chamberlain et al. 1997) and TGFβ (Beebe et al. 2004) are critical for the development of the lens. Another class of molecules that has been proposed to control lens development is the β1-integrins (Menko et al. 1998; Bassnett et al. 1999; Walker and Menko, 1999), a family of cell surface receptors formed by heterodimerization of a β1 subunit with 12 different α subunits. Integrins are expressed in many tissues where they anchor cells to the surrounding ECM, including basement membranes (Gianotti and Ruoslahti, 1999; De’Arcangelis and Georges-Labouesse, 2000; Hynes, 2002). Basement membranes (BM) are sheet-like ECM structures at the junction of epithelial, muscle and endothelial cells and their respective connective tissues (Timpl and Brown, 1996).

Integrin-mediated binding of cells to the ECM activates cellular signal transduction cascades (Gianotti and Ruoslahti, 1999; Hynes, 2002; Li et al. 2003). Signaling is achieved by the integrin-dependent recruitment of cytoplasmic proteins to adhesion sites. One integrin effector is integrin linked kinase (ILK), which binds to the integrin β1 cytoplasmic domain and recruits additional proteins such as Pinch and Parvin to establish a link between integrins and the cellular cytoskeleton (Dedhar, 2000; Wu, 2005). Genetic studies in mice have shown that both β1-integrin and ILK knockouts result in early embryonic lethality (Fassler and Meyer, 1995; Stephens et al. 1995; Fassler et al. 1996; Sakai et al. 2003). Viable conditional knockouts have revealed an essential role of β1 in stability of the pial BM, as well as the regulation of lamination in the cortex and cerebellum, and the formation of the neuro-muscular junction (Georges-Labouesse et al. 1998; Graus-Porta et al. 2001; Schwander et al. 2004), with a similar CNS phenotype found in the ILK conditional knockouts (Feltri et al. 2002; Belvindrah et al. 2006).

Several integrin subunits such as α5, α6, β1 and β3 are expressed in the lens where they are proposed to anchor anterior lens epithelia and lens fibers to the lens capsule (Menko and Philips, 1995; Menko et al. 1998; Walker et al. 2002; Zelenka, 2004; Wederell et al. 2005), and be essential for developing fiber growth and migration (Menko et al. 1998; Zelenka, 2004; Wederell et al. 2005). To investigate the importance of integrins in lens fiber adhesion and development we investigated the lens phenotypes of nestin-restricted β1-integrin and ILK knockouts. Using CRE/LOX mediated gene ablation, we provide evidence that β1-integrin and its downstream signaling partner ILK play an important role in migration, postnatal lens fiber survival and in the assembly and maintenance of the lens capsule. The similar phenotypes of β1 and ILK mutants suggest that both proteins work in concert in these processes.

## Results

### β1-integrin expression in lenses of nestin-Itgb1Ko mice

**Figure 1:**
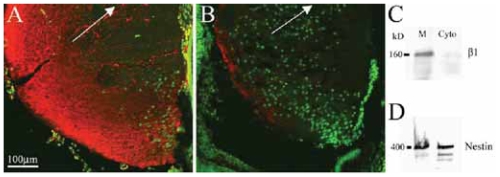
β1-integrin expression in control and mutant lens. Side-by-side comparison of a P1 control (**A**) and mutant (**B**) lens cross-section confirmed that β1-integrin expression (red) in the P1 mutant lens was nearly eliminated. The nuclear counter-stain (green) showed the abnormal location of nuclei in the posterior pole of the mutant lens (**B**). Western blots confirmed that β1-integrin (**C**) and nestin (**D**) were present in the developing P1 lens of control mice. β1-integrin was detectable in the membrane (M) but not the cytoplasmic fraction (Cyto) of the lens, nestin was detected in both fractions. The arrows point towards the anterior region of the lens and are aligned along the lens midline.

To confirm the expression of the integrin β1 subunit gene (*Itgb1*) in the developing mouse lens, we labeled E18 and P1 mouse lenses with anti-β1-integrin antibodies and established strong labeling of lens fibers (Fig. 1A and data not shown) consistent with previous work (Wederell et al. 2005). Western blots further established the presence of β1-integrin in the control lens. As expected, the β1-integrin protein band was abundant in the plasma membrane fraction of lens fibers and almost undetectable in the cytoplasmic fraction (Fig. 1C). Complete knockout of the *Itgb1* gene in mice leads to early embryonic lethality prior to eye formation (Fassler and Meyer, 1995; Stephens et al. 1995). To study the function of the *Itgb1* gene in the lens, we took advantage of *Itgb1-CNSko* mice. These mice were obtained by crossing mice carrying an *Itgb1-flox* allele with *nestin-Cre* mice and are viable into adulthood (Graus-Porta et al. 2001; Blaess et al. 2004). Nestin is an intermediate filament that is particularly prominent in the developing central nervous system (Lendahl et al. 1990), but is also expressed in the lens (Yang et al. 2000). Expression of nestin in lens fibers was confirmed through western blot analysis showing a strong band of nestin protein in the membrane and cytoplasmic fractions of control lens homogenates (Fig. 1D), therefore indicating that nestin is appropriately located to mediate recombination of the *Itgb1* gene in the lens. Immunohistochemistry revealed that the β1-integrin protein was greatly reduced in the mutant lenses (Fig. 1B), but was abundant in the heterozygote sibling (Fig. 1A) with highest concentrations in regions closest to the lens capsule. Residual integrins in the lens generated prior to Cre-recombination may explain the appearance of a slightly incomplete knockout. Western blot detection of ILK in control lens homogenates was considered unsuccessful due to the failure of antibodies to specifically detect ILK in lens tissue; the labeled band at approximately 50–60kD was too weak to make an unequivocal positive ID. Similarly, detection of ILK by immunohistochemistry was inconclusive because of high background labeling.

### Lens phenotype in β1-integrin mutant mice

**Figure 2:**
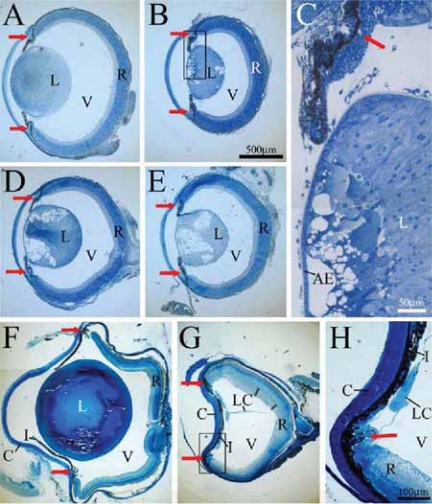
Ocular histology of β1-mutant and control mice. Cross-sections of control (**A**, **F**) and β1-mutant mice (**B**–**E**,**G**,**H**) at P1 (**A**–**E**) and P60 (**F**–**H**) showed early and late defects of the lens of β1 integrin-defective mice. Higher magnification of the selected areas in (**B** and **G**) are shown in (**C** and **H**) respectively. Initial stages of lens degeneration were indicated by lens fiber vacuolization in P1 mutants (**B**–**E**) however ciliary body and anterior epithelial structures remained normal (**C**). By P60, normal lens structures (**F**; control) were absent in the mutant mice (**G**). Note the fusion of iris and cornea indicating a loss of the anterior chamber. Remaining lens capsule strands could be seen next to the iris and ciliary body (**H**). **Abbreviations:** AE, anterior epithelium; L, lens; V, vitreous; R, retina; I, iris; C, cornea; LC, lens capsule. The red arrows indicate the ciliary body structures.

The development of lenses was compared between *Itgb1-CNSko* mice and heterozygous siblings. Cross-sections through P1 mutant (Fig. 2B–E; n = 6) and control eyes (Fig. 2A; n = 6) revealed morphological differences, most obvious were alterations in the lenses of mutants. All mutant lenses showed vacuolized areas accompanied by a shape change from ellipsoid in control eyes to an almost cuboidal shape in the mutants (compare Fig. 2B–E and 4B, D with 2A and 4A, C). Despite lens fiber abnormalities, the anterior lens epithelium and the ciliary body both appeared normal (Fig. 2C).

By P60, heterozygous and non-mutant mice developed very large lenses (Fig. 2F; n = 8), whereas all of the mutant eyes were aphakic (Fig. 2G; n = 8). In most cases sparse remnants of lens-like material could still be detected in the mutant eye (Fig. 2G, H). In all P60 mutants the iris was fused with the cornea (Fig. 2H).

**Figure 3:**
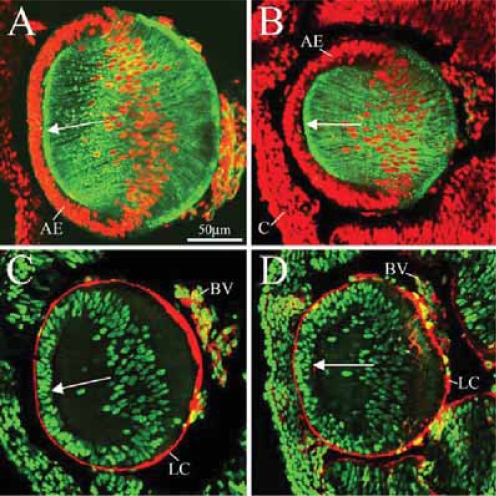
Nuclear localization, lens fiber morphology and lens capsule structure in E13 control (**A**, **C**) and mutant (**B**, **D**) specimens. Cross-sections were stained for phalloidin (green; lens fiber morphology) and nuclei (red; **A**, **B**), or stained for collagen IV (red; lens capsule) and nuclei (green; **C**, **D**). In both mutant and control E13 lenses, normal nuclear distribution is found in both the anterior epithelium and posterior lens fibers (**A**–**D**). The lens fiber morphology (**A**, **B**) and lens capsule structure (**C**, **D**) was similarly normal in both controls and mutants at E13. Arrows point to the anterior pole of the lenses. **Abbreviations:** AE, anterior epithelium; BV, blood vessels; C, cornea; LC, lens capsule.

To better understand the developmental etiology of this mutant phenotype we first looked at how this mutation manifests at an earlier embryonic stage around the time of recombination. Yang et al. (2000) found that nestin protein expression appeared in posterior lens epithelium at 10.5 days post coitus (dpc) with expression in lens fiber transcripts by 14.5 dpc. Embryonic day 13 embryos were assessed to approximate a nestin-mediated peri-recombination time point. We analyzed and compared mutant and control lenses along several criteria: distribution of lens epithelial and lens fiber nuclei, changes in cell shape and cytoskeletal organization of lens fibers, and status of the lens capsule. The morphology of lens fibers was visualized by staining for F-actin by using fluorescent phalloidin as a probe (Bassnett et al. 1999), and the status of the lens capsule was established by staining for collagen IV, a prominent component of basement membranes. At E13 there are no apparent defects in the lens of the conditional knockout. In both E13 control (n = 4) and mutant lenses (n = 4) nuclear distribution is normal along both the anterior epithelial and equatorial (lens fiber) regions (Fig. 3A–D). Lens fiber morphology in the E13 mutant (Fig. 3B) appears neatly arranged similar to the E13 control lens (Fig. 3A). A robust and unfragmented lens capsule was found in both the mutant (Fig. 3D) and control (Fig. 3C) lenses. Thus, it appears up until recombination β1 mutants display normal lens formation.

**Figure 4:**
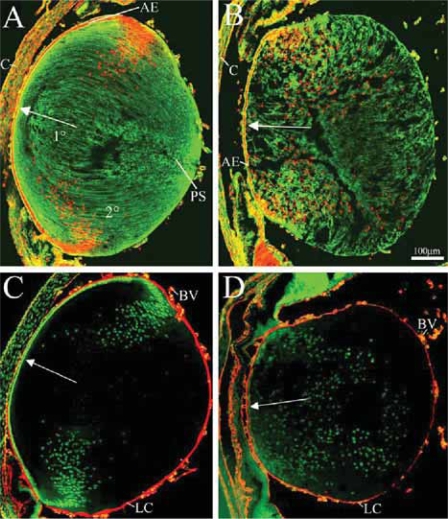
Lens capsule and lens fiber disruption at P1. Cross-sections of control (**A**, **C**) and mutant lenses (**B**, **D**) were stained for either phalloidin (green; and nuclei — red; **A**, **B**), or collagen IV (red; and nuclei — green; **C**, **D**) to show lens fiber morphology and lens capsule structure (respectively). In P1 control lenses (**A**, **C**), a monolayer of cells formed the anterior lens epithelium. The lens fiber nuclei were neatly arranged around the equator of the lens, and the lens capsule was well delineated and continuous. In the P1 β1-mutant lenses (**B**, **D**) nuclei were randomly distributed throughout the lens and the lens capsule appears fragmented. The anterior epithelium, however, appeared fairly normal. Arrows point to the anterior pole of the lenses. **Abbreviations:** 1°, primary lens fibers; 2°, secondary lens fibers; AE, anterior epithelium; BV, blood vessels; C, cornea; LC, lens capsule; PS, posterior seam.

By P1, however, wild-type and mutant lenses differed greatly along these same criteria. We found in control lenses at P1 (Fig. 4A) the typical lens epithelium monolayer in the anterior part of the lens, and the bow-shaped nuclei at the equatorial region where lens epithelial cells differentiate to become the long, denucleated secondary lens fibers. In mutants at P1, cell nuclei of lens fibers were found in almost all areas of the lens yet the monolayer of anterior lens epithelial cells remained intact (Fig. 2C, 4B, D).

To find out whether integrin deletion lead to a change in lens fiber morphology we again compared mutant and control. Phalloidin labeling revealed the long lens fibers in P1 control lenses (Fig. 4A). The staining also revealed the central core of primary lens fibers, the outer layers of secondary lens fibers and the anterior and posterior seams, where the secondary lens fibers interdigitize (Fig. 4A). In P1 mutant lenses, the basic organization of lens fibers was perturbed (Fig. 4B). It was evident that the migration of secondary fibers was greatly disrupted as they failed to interdigitize resulting in a loss of both anterior and posterior seams. It is of note that the primary lens fibers did not all lose their nuclei as they would normally do (Fig. 4B).

Immunohistochemical investigation of collagen IV in P1 lenses revealed changes in the lens capsules of mutant mice. In controls, the basement membrane (BM) of the lens appears robust and continuous (Fig. 4C). Attenuation of the lens capsule was common in P1 mutants (Fig. 4D) with multiple disruptions in lens capsule continuity. By P6, the BM integrity is further compromised with frequent disruptions along all regions of the lens capsule, whereas by P60 only lens capsule fragments remain in the absence of a lens (data not shown).

**Figure 5:**
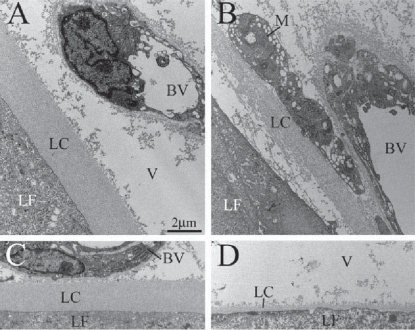
Electron micrographs showing defects in the lens capsule of P1 β1-mutant mice. The lens capsule in P1 control mice was between 2 and 3 μm thick and the lens fibers were tightly attached to the capsule (**A**). The lens capsule from the posterior pole of a normal lens (**C**) was thinner than the capsule at the equatorial and anterior pole of the lens. In the mutant mice (**B**, **D**), the lens capsule showed signs of disintegration and lens fibers detachment (**B**) Macrophages were often seen in close proximity of the degenerating lens. The capsule from the posterior pole of the mutant lens was unusually thin (**D**) as compared to the lens capsule from a corresponding site of a control eye (**C**). **Abbreviations:** BV, blood vessel; M, macrophage; LC, lens capsule; V, vitreous; LF, lens fibers.

Due to our immunohistochemical results at this stage we used electron microscopy to further investigate the ultra-structure of the lens BM. The lens capsule in P1 controls appeared as a 2 to 3 μm thick BM that was thicker at the anterior and equatorial poles of the lens (Fig. 5A), and thinner in the posterior pole (Fig. 5C). As expected from immunolabeling, the lens capsule in the P1 mutant eye was thinner than normal. Separation of the lens capsule was observed in equatorial and posterior poles of the lens with exceptional thinning noted in the posterior pole (Fig. 5B and 5D respectively, and data not shown). We frequently detected the presence of macrophages close to the degenerating lens (Fig. 5B). Our investigations showed that the deletion of β1-integrin in lens leads to a redistribution of lens nuclei, a change in fiber morphology, disruption of the lens capsule and a striking loss of lens tissue. In order to determine if integrin-mediated intracellular signaling is an important factor in these changes, we analyzed the lens phenotype in a mouse with a conditional deletion for ILK, one of the central signaling molecules in the integrin signaling pathway.

### Lens phenotype in ILK mutant mice

**Figure 6:**
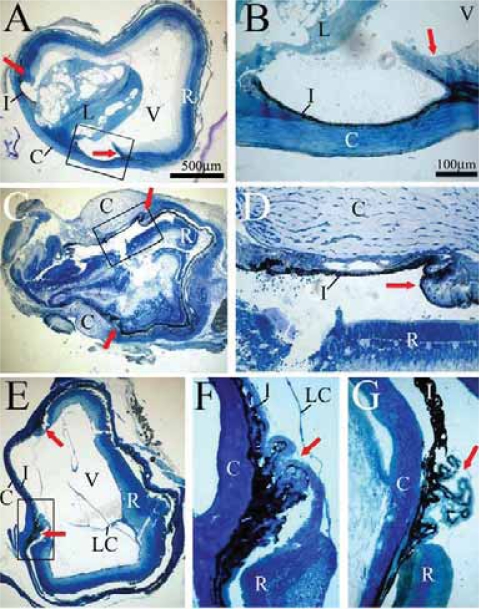
ILK-mutant and control ocular histology. Cross-section of both eyes of a P10 ILK-mutant mouse (**A**–**D**), showed massive degeneration of the lens. High power micrographs of the selected areas in (**A**) and (**C**) are shown in (**B**) and (**D**), respectively. In the eye shown in (**C**), the lens ruptured and was ejected through the cornea. Eyes from P60 ILK-mutant mice were consistently aphakic (**E**). A high power view of the selected area is shown in (**F**). ILK-mutants also displayed a fusion of the iris and cornea (**B**, **D**, **F**) compared to the nicely separated structures in the P60 control (**G**). A few threads of lens capsule were evident in the vitreous cavity (**F**). The red arrows points to the ciliary body. **Abbreviations:** C, cornea; I, iris; L, lens; V, vitreous; R, retina; LC, lens capsule.

ILK is a cytoplasmic serine-threonine kinase that plays a central role in integrin-dependent signaling (Hannigan et al. 1996). Sections through P10 mutant eyes (n = 4) showed that the lenses experienced dramatic changes: in one case, the majority of lens fibers had degenerated and half of the lens consisted of vacuoles (Fig. 6A, B). In this case, both irises had fused with the cornea and part of the lens was in direct contact with this corneal/iris complex (Fig. 6B). The contralateral eye (Fig. 6C) of the same animal displayed a complete rupture and delocalization of the lens. Consistent with the contralateral eye and analogous phenotypes in the β1-mutant, the iris had again fused with the cornea (Fig. 6D). Four other eyes from the same stage of development showed a very similar morphology as shown in Figure 6C, where the lens had been entirely lost. In all cases, the cornea was centrally perforated, suggesting that the lens had ruptured and was extruded through a central hole in the cornea.

At P60, all mutant eyes were aphakic (Fig. 6E), or were greatly disrupted (similar to that shown in Fig. 6C; n = 4). Greater magnification revealed iris/corneal fusion, a normal ciliary body, and a few detectable lens remnants (Fig. 6F). Analogous sections of P60 control eyes showed normal separation of iris and cornea (Fig. 6G).

**Figure 7:**
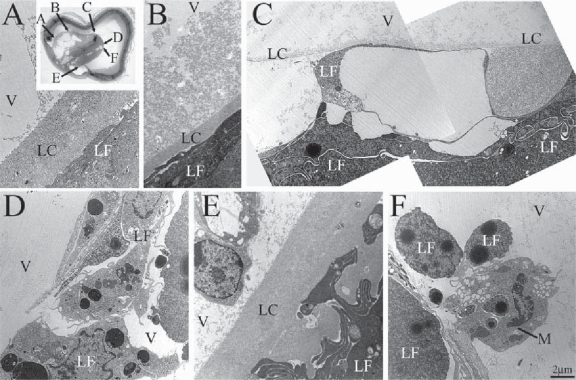
Electron micrographs showed defects in the lens capsule of P10 ILK-mutant mice. In the equatorial parts of the lens (**A**, **E**), the lens capsule was thick and resembled control eyes (**A**), yet in many areas the capsule invaded the lens tissue (**E**). Posterior to these regions, thinning of the lens capsule was seen accompanied by a cloud of lens capsule debris (**B**). In the posterior pole of the lens (**C**, **D**, **F**), the lens capsule was either very thin (**C**) or entirely absent (**D**, **F**). In areas, there was a gross separation of the lens capsule and lens fibers (**C**). Degeneration of adjacent lens fibers was frequently seen (**C**, **D**), as well as macrophage ingestion of degenerating lens particles (**F**) and vitreal invaginations of the lens (**D**). **Abbreviations:** V, vitreous; LC, lens capsule; LF, lens fibers; M, macrophage.

Electron microscopy of the ILK-mutant P10 lens capsule revealed a range of lens capsule and lens fiber disruptions. These ranged from control-resembling, thick lens capsule portions in the anterior pole (Fig. 7A) to areas of moderate lens capsule disruption, similar to that seen in the P1 β1-mutants, in the equatorial pole (Fig. 7B, C) to areas of severe lens capsule and fiber disruption in the posterior pole (Fig. 7C, D, F). Though most anterior lens capsule structures appeared normal, in many places the lens capsule displayed aberrant extensions into the lens fibers (Fig. 7E). Thinning (Fig. 7B) and withdrawal (Fig. 7C) of the lens capsule was observed in the equatorial poles of the lens. In more posterior regions the lens capsule was either very thin (Fig. 7C) or entirely absent (Fig. 7D, F). Separation of adjacent lens fibers, accompanied by vitreal invaginations of the lens, was observed in portions of the posterior lens (Fig. 7D). We also localized numerous macrophages close to the degenerating lens, some of which even contained parts of the degenerated lens fibers within their phagosomes (Fig. 7F).

**Figure 8:**
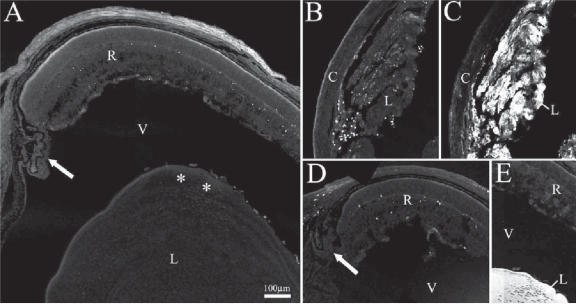
Apoptosis in control and ILK mutant eyes. TUNEL staining in a P7 control mouse eye (**A**) revealed no apoptosis in the lens. Apoptosis was found in lens fragments of P7 ILK mutants (**B**). These fragments were confirmed to be lens remnants through immuno-staining for α-crystallin (**C**). P7 ILK retina (**D**) exhibited low levels of tonic apoptosis similar to control retina (**A**). The ciliary bodies (arrows) of controls and mutants (**A**, **D**) showed no apoptosis. Crystallin staining (**E**) of control eye in (**A**) reveals robust staining of lens (lens region indicated by “*” in A). **Abbreviations:** C, cornea; L, lens; V, vitreous; R, retina.

To address the mechanism of lens degeneration, TUNEL staining was performed on P7 ILK mutants to investigate apoptotic cell death. Control P7 lenses revealed no apoptosis in the lens (Fig. 8A) whereas ILK mutant lens fragments showed ubiquitous cell death with regions of stronger staining (Fig. 8B). Apoptotic levels in the retina and ciliary body were similar amongst WT and mutants (Fig. 8A, D) with scattered cell death in the retina and no cell death in the ciliary bodies. In order to identify remaining lens fragments in the P7 ILK mutant, crystallin staining was performed on control and mutant sections. Lenses of control sections fluoresced brightly in response to crystallin staining with no expression found in other ocular areas (Fig. 8E). Likewise, apoptotic fragments in Figure. 8B fluoresced brightly when similarly stained (Fig. 8C).

## Discussion

### Nestin-Cre-dependent deletion of β1 integrin in lens

The current study shows that ECM receptors, the β1-integrins, are required for proper lens fiber development. Consistent with earlier studies in the lens, we found that β1-integrin was expressed (Fig. 1A, C) (Menko and Philips, 1995; Walker and Menko, 1999; Walker et al. 2002; Wederell et al. 2005), nestin was expressed (Fig. 1D) (Yang et al. 2000) and nestin-linked Cre can be used to delete gene expression in the lens (Robinson et al. *IOVS* 2006; 47:ARVO E-Abstract 1101).

Nestin mRNA appeared in differentiated lens fibers by 14.5 dpc (Yang et al. 2000). The nestin-linked β1-integrin deletion in our mutant mice likely occurred in close temporal proximity to the onset of nestin expression in the lens. Following the deletion, it is possible that with continued lens growth the remaining integrins on existing fibers were increasingly inadequate, with newly generated fibers lacking β1-integrin entirely. This might help explain the increasing pathology with increasing age (Fig. 2–4).

### Deletion of β1 and ILK lead to disruption and degeneration of lens

The phenotypes observed in β1 and ILK mutants could result from loss or attenuation of the lens capsule, integrin-mediated cell adhesion difficulties, changes in downstream signaling (e.g. disruption in survival pathways), or some combination of the three.

#### Attenuation of lens capsule

A consistent feature among mutants was the extreme thinning or even absence of the lens capsule in the posterior lens. The lens capsule, like all other BMs, is composed of members of the laminin family, collagens IV and XVIII, perlecan and nidogen (Cammarata et al. 1986; Dong et al. 2002). Mutation of the ECM protein laminin-1 leads to severe lens defects in zebrafish (Gross, et al. 2005; Zinkevich et al. 2006; Lee and Gross, 2007) with adults displaying strikingly similar phenotypes to our β1-integrin and ILK mutants including aphakia, lens cellular disorganization and reduced eye size. The similar lens phenotype following laminin and integrin deletion suggests a functional connection of extracellular matrix and an integrin-mediated intra-cellular signaling cascade. Additionally, the ECM protein collagen IV appears to maintain lens survival through protection of lens epithelial cells from Fas-dependent apoptosis (Futter et al. 2005).

Though the protective roles of the lens capsule are important, the loss of ECM proteins in our mutants is temporally secondary to the loss of β1-integrin and ILK. It has been well established that integrins control and promote BM assembly (Schwartz et al. 1995; Wu et al. 1995; Fassler et al. 1996; Ruoslahti, 1996; Aumailley et al. 2000; Li et al. 2002; Mao and Schwarzbauer, 2005). Also, integrin deletion has previously been found to result in a failure of BM assembly: BMs that separate the epidermal from the endodermal cell layers of the morula fail to constitute as a result of β1-deletion leading to inner cell mass apoptosis (Fassler and Meyer, 1995; Stephens et al. 1995). Similar to β1-integrin, there is evidence that ILK positively regulates ECM assembly (Wu et al. 1998; Vouret-Craviari et al. 2004). As our mutants aged from the time of the deletion, the lens capsule changed from a thick to a thin basement membrane structure. With the loss of integrin function, it is possible that the lens fibers were unable to recruit new ECM proteins to account for the growing lens size, therefore straining the lens capsule and resulting in the characteristic lens capsule thinning (Fig. 4, 5).

Though a (loss of ) lens capsule-mediated phenotype is possible, the presence of ubiquitous vacuolization and cellular reorganization in these mutants, while the lens capsule remains largely intact, indicates that lens fiber apoptosis probably does not occur as a result of lens capsule-mediated cell death. However this may contribute to the accelerated pathology seen shortly thereafter.

#### Lens fiber adhesion and migration

Correct lens fiber morphology is proposed to be established through integrin-ECM protein mediated lens fiber migration (Ruoslahti, 1996; Zelenka, 2004). We have shown here that loss of integrin function significantly impaired migration as evident by the loss of the anterior and posterior seams (Fig. 4B). The disrupted morphology in β1 mutants may have resulted from integrin-mediated adhesion difficulties between lens fibers and their ECM guides.

Integrins can participate in certain cell-cell adhesions in vertebrates (Hynes, 2002), however it is unclear if integrin-mediated cell-cell interactions occur in the lens. Interestingly, upon maturation secondary lens fibers break contact with the lens capsule (Kuszak et al. 2004), therefore only the most superficial, recently developed or developing fibers will have integrin-ECM protein connectivity. If a deficit in this connectivity were to explain the cell loss in β1-integrin mutants, then one would expect an “outside-in” pattern of cell death, initially targeting peripheral fibers. However, death of central fibers lacking a BM substrate in our mutants (Fig. 2, 4) indicates a potential novel role of β1-integrin in cell-cell interactions in the normal lens. This also indicates that disrupted lens morphologies could result from loss of adhesion between adjacent fibers.

#### Altered integrin signaling

Integrins participate in signaling across the plasma membrane (Hynes, 2002). ILK is an intermediate of integrin signaling and is involved in activating PKB/AKT in response to integrin binding, an interaction that is required for cell survival (Persad et al. 2000; Troussard et al. 2003; Friedrich et al. 2004). The deletion of ILK resulted in an early perinatal loss of lens fibers very similar to β1 deletion suggesting that a disruption in downstream integrin signaling is the cause of lens degeneration. In some cases, an earlier and more severe phenotype was observed in ILK mutants (Fig. 6C, 7 and data not shown). ILK binds several integrin family members including β1 and β3 (Wu and Dedhar, 2001), and we postulate that this is the cause of the more severe ILK phenotype.

One likely candidate for signaling-mediated cell death is anoikis, i.e. a loss-of-substrate mediated apoptosis (Frisch et al. 1996; Zhan et al. 2004). Anoikis is mediated by the disruption of integrin binding or the interference of the down-stream signaling molecules, including ILK and the survival factors PKB/Akt (Zhan et al. 2004). ILK prevents anoikis through phosphorylation and activation of these survival factors (Persad et al. 2000; Troussard et al. 2003). Though immunohistochemical investigation of this pathway was not informative (Data not shown) we hypothesize that this was the most likely mechanism of cell death experienced by the majority of lens fibers.

### Lens fiber cell death

Though the intermediate steps involving lens fiber degeneration remain unclear, the end result appears to be apoptosis. TUNEL staining on P1 β1 mutants revealed faint punctate regions of apoptotic cell death (Data not shown). In older, more severely affected mutants greater apoptosis was found (Fig. 8). ILK P7 mutants were ideal since massive degeneration was present at this stage but the eyes are not yet completely aphakic. In addition, macrophages have been implicated with removal of apoptotic dead cells in the lens (Garcia-Porrero et al. 1984; Nishitani and Sasaki, 2006). Numerous macrophages were found adjacent to integrin and ILK mutant lens fibers and lens capsule (Fig. 5, 7) with few or no macrophages found in controls.

### Mutant lens phenotype is primarily due to lens fiber defects

At P1, when vacuolization and lens fiber defects were apparent, the anterior epithelium was not yet disturbed (Fig. 2, 4) indicating that defects in the anterior epithelium occurred secondarily to those in lens fibers and therefore were not primarily involved in the mechanisms of the mutant lens pathology. Similarly at P1, the ciliary body and retina appeared normal (Fig. 2) even though nestin is expressed in retinal neuroepithelium (Yang et al. 2000). Apoptosis and organization of the retina and ciliary body were similarly normal in the P7 ILK mutant (Fig. 8 and 6 respectively). Adult, P60 β1 and ILK mutants (Fig. 2G, H and 6E, F) show these same structures to be intact suggesting that the lens phenotype is likely the result of lens fiber defects and not defects elsewhere in the eye.

### Integrins and congenital cataracts

In contrast to frequent age-related cataracts, congenital cataracts are a rare condition in which the lens degenerates in early childhood. Some of the congenital cataracts stem from mutations of transcription factors such as Pax6, Pitx3, Eya and others (Graw, 2004). The down-stream target proteins of these transcription factors, eventually responsible for the lens defects in congenital cataracts, are unknown. Interestingly knockout of the Foxe3 gene, a downstream target of Pax6 (Dimanlig et al. 2001), results in a similar lens pathology as our β1 and ILK mutants (Medina-Martinez et al. 2005). Also, over-expression of Pax6 expression in the mouse lens results in augmented levels of α5β1-integrin and subsequent early, postnatal cataracts (Duncan et al. 2000). The aphakia seen in congenital cataracts may share similar mechanisms to those contributing to the aphakia in our β1 and ILK mutants. We propose that disruption of integrin signaling is potentially responsible for some of the congenital cataract cases.

## Experimental Procedures

### Mice

*Nestin-CRE* mice and mice carrying floxed alleles for the *Itgb1* and *ILK* genes have been described previously (Tronche et al. 1999; Graus-Porta et al. 2001; Terpstra et al. 2003). The production and screening of the mice for homozygous mutations have been similarly described (Graus-Porta et al. 2001; Blaess et al. 2004; Belvindrah et al. 2006).

### Histology

Eyes were fixed in 4% paraformaldehyde overnight, sunk in sucrose and crysostat sectioned at 12 μm after embedding in Tissue-Tek (OCT compound; VWR International, Bridgeport, NJ). Sections were blocked with 0.1% Triton-X-100/2%BSA in PBS. Primary antibodies were: a polyclonal antiserum to β1 integrin (Schwander et al. 2004), a polyclonal to collagen IV (Rockland, Gilbertsville, PA), and a polyclonal to α A crystallin (Santa Cruz Biotechnology, Santa Cruz, CA). Antibodies to ILK included a polyclonal antiserum raised against a synthetic C-terminal peptide (Cell Signalling technologies, Danvers, MA) and 2 mono-clonal antibodies (Belvindrah et al. 2006). Lens cells were labeled with biotin-Bandeiraea simplifolia isolectin IIB (Vector Laboratories, Burlingame, CA; not shown) and with Oregon Green phalloidin (Molecular probes, Eugene, OR) each at a dilution of 1:100. Secondary antibodies and fluorescent labels were Cy3-labeled Goat anti-mouse, goat anti-rabbit antibodies and Cy3-labeled streptavidin (Jackson Immuno Research, West Grove, PA). Sytox Green or Sytox Orange (Molecular Probes) were used as nuclear counter stains. TUNEL staining on frozen sections was performed using the DeadEnd Fluoromeric TUNEL System (Promega Corporation, Madison, WI). The sections were photographed using an Olympus confocal microscope.

For electron microscopy and semi-thin sectioning, the eyes were fixed in a 2.5% glutaraldehyde, 0.1% picric acid and 0.02% tannic acid cocktail overnight. Osmication and embedding in EPON followed standard procedures. For light microscopy, the embedded eyes were sectioned at 2 μm. Sections were spread onto Superforst/Plus slides (Fisher Scientific) and stained with each 0.5% methylene blue/azur II in 0.5% borax at 65 °C for 80 seconds (Richardson et al. 1960). Thin sections for EM were stained with uranyl acetate and lead citrate and viewed with a JEOL-JEM-1011 electron microscope.

### Western blotting

Lenses from 6 P1 mice were homogenized in PBS and centrifuged at 10,000 rpm to separate membrane and nuclear fractions from the cytoplasm. Samples from both fractions were run out by SDS PAGE, the proteins transferred to nitrocellulose and the blots labeled with anti-β1-integrin, anti-ILK and anti-nestin (rat 401; Dev. Studies Hybridoma Bank, University of Iowa). The proteins were visualized by alkaline phosphatase-labeled goat-anti-rabbit and goat-anti-mouse IgG (Jackson ImmunoResearch) followed by NBT/BCIP (Roche, Indianapolis, IN) staining.
